# The Practice of Surgery

**Published:** 1857-11

**Authors:** 


ART. X.—*The Practice of Surgery.* By JAMES MILLER, F.R.S.E., F.R.C.S.C., Surgeon in Ordinary for the Queen for Scotland; Surgeon in Ordinary to his Royal Highness Prince Albert for Scotland; Professor of Surgery in the University of Edinburgh; Consulting Surgeon to the Royal Infirmary, &c. &c. Revised by the American Editor. Fourth American, from the last Edinburgh Edition. Illustrated by three hundred and sixty-four Engravings on Wood. 8vo. pp. 682. Philadelphia: Blanchard & Lea. 1857.

OF a work so universally known as this, it is not necessary to say anything further than simply to announce the publication of a new edition. A formal introduction to the profession of this country would be as absurd as a formal introduction of a child to its parent. There is hardly a respectable practitioner in the United States who is not practically conversant with its merits. Excepting the work of Professor Fergusson, of London, so well and so favorably known to our readers, we are not acquainted with any treatise on practical surgery in the English language at all comparable to that of Mr. Miller. As an accurate, faithful, and graphic nosographer, we have long regarded the author as without a rival. His style, without being always elegant, is eminently vigorous and impressive, and altogether *Millerian* in its character. It will be recollected by our readers that the edition of this work immediately preceding this, was edited by Dr. Sargent, of this city, but as this gentleman has been for some time absent in Europe, the present issue has been brought out under the superintendence of one of his friends, whose name, however, does not appear upon the title-page, nor, indeed, in any other part of the work. He states, in his preface, that he has "added some additional matter," chiefly in relation to the progress of surgery in this country; and the numerous and valuable references which he has made to American Journals, bear him out in the statement. The most important of these are an account of Dr. Bozeman's operation for vesico-vaginal fistule, first published in the Louisville Review, and subsequently in this Journal. He has also added a number of new illustrations, which somewhat enhance the value of the work. The new edition is issued in a style highly creditable to the eminent publishers.

